# Searching for a Soul

**Published:** 1889-08

**Authors:** 


### SEARCHING FOR A SOUL.

Materialism cannot furnish a more complete exemplification of its spirit and meaning than it did in the case of the three doctors who sought to discover the secret power of Bishop, the noted mind-reader, at the point of the scalpel in the hands of the operator. They adroit ly went at their work before the soul could fairly be supposed to have left the body, apparently in search of the soul itself, as if to question it concerning the mystery of its lodgment and the secret of its control. It might perhaps be regarded as a case of vivisection for the discovery of the spirit before it had taken its leave.

They could have sought for nothing more than material evidences of the spiritual functions in the human body, and thus far, though the circumstances were unparalleled in autopsical history, it has not been disclosed that their strange efforts were rewarded with the least success. They made one thing certain, that the man was dead when they got through with him, which is not generally believed to have been the case when they took him in hand.

The preposterous thing about it is that they should have expected to discover the secret of the phenomenon exhibited by Bishop by an examination of material organs of his body. If they did not expect that, then why make the autopsy at all, and why, of all other things, make it in such haste as to horrify every one who has heard of their conduct? It is a clear case of materialism probing for the living principle which mere materialism need never hope to find. The method pursued, too, was strictly consistent with materialistic theories. It assumed that in spirit the principle of all life is something that can be handled, measured, weighed and treated after a material fashion.

But, happily for us all, life is something that can neither be comprehended nor investigated in a material way. It is not less real either, because it is so utterly elusive of sense. Man is not so low and mean a being as the animal the materialists believe him to be. There are facts in man's existence which none of the so-called scientific theories can ever hope to explain. The materialists might as well try to see with the ear and hear with the eye as uncover the spirit with the scalpel. And a person blind from birth might as well undertake to deny the existence of light and color as materialists to deny the existence of a soul.—*Boston Globe.*

